# Assessing the Association of Healthcare Resource Utilization and Patient-Reported Outcomes on Shared Decision-Making among Multimorbid Individuals

**DOI:** 10.3390/healthcare12171709

**Published:** 2024-08-27

**Authors:** Syeda Hina Zaidi, David Rhys Axon

**Affiliations:** Department of Pharmacy Practice and Science, College of Pharmacy, The University of Arizona, 1295 North Martin Avenue, P.O. Box 210202, Tucson, AZ 85721, USA; draxon@arizona.edu

**Keywords:** shared decision making, multimorbidity, healthcare resource utilization, patient reported outcomes, chronic disease

## Abstract

Shared decision-making (SDM) is an essential component of patient-centered healthcare and disease management. However, the association of SDM with healthcare resource utilization and patient-reported outcomes among multimorbid individuals is not well understood. This study sought to evaluate the association of SDM with healthcare resource utilization and patient-reported outcomes among United States (US) adults with multimorbidity. Data were collected from the 2020 Medical Expenditure Panel Survey (MEPS) for this cross-sectional study. Eligible participants were US adults with two or more comorbidities. The predictor variable was SDM (optimal versus not optimal). The outcome variables were healthcare resource utilization and patient-reported outcomes. Logistic regression models, adjusted for demographic characteristics, assessed associations with SDM for each healthcare resource utilization and patient-reported outcome variable. The analysis maintained the complex survey data and was weighted to produce nationally representative estimates. Individuals who reported optimal SDM in adjusted analyses utilized more healthcare resources compared to those who reported not optimal SDM. Individuals with optimal SDM had more than one outpatient visit (odds ratio OR = 1.23, 95% CI = 1.03–1.47), emergency room visit (OR = 1.55, 95% CI = 1.17–2.06), and inpatient discharge (OR = 1.44, 95% CI = 1.05–1.96). Additionally, these individuals had higher odds of reporting limitations in their ability to work or engage in other activities due to their physical health in the past four weeks (OR = 1.27, 95% CI = 1.01–1.60). This study indicated evidence of increased healthcare resource utilization among patients who participate in SDM with their providers, which should be explored in future studies.

## 1. Introduction

Multimorbidity, defined as the co-occurrence of two or more chronic conditions in the same individual [1], is a considerable public health issue. The high prevalence of multimorbidity is associated with negative health and societal consequences such as increased mortality rates, functional limitations, healthcare utilization, and healthcare costs that can impact a person’s overall well-being and quality of life [2]. Clinical guidelines for multimorbidity emphasize the need to shift from a single condition focus to a multimorbidity approach, addressing patient priorities and creating individualized care plans. Evidence for treating multimorbidity is limited, with most trials excluding patients with multiple chronic conditions, highlighting the need for more inclusive research [3]. Access to information, coordination of services, and focus on patient-centered approaches are a priority on the list of healthcare needs for patients with multimorbidity [4]. Research using semi-structured, in-depth interviews with general practitioners revealed patient-centered care as the most promising approach to managing multimorbidity [5]. Given the complexity of managing multiple chronic conditions, there is a growing recognition of the need for patient-centered approaches like shared decision-making (SDM). 

SDM is defined by Elwyn et al. as “an approach where clinicians and patients make decisions together using the best available evidence” [6]. The implementation of SDM has the potential benefit of improving health outcomes through the more active involvement of patients. However, the effect of SDM in improving healthcare utilization and health outcomes in patients with multimorbidity can be complicated because of the decreased participation of these patients in the decision-making process and single disease-based guidelines for physicians. In addition, more than one health professional is often involved in the management of chronic conditions, which can lead to conflicting views on treatment priority and patient involvement [7]. 

Multiple models are available to evaluate SDM, with the three-talk model and the SHARE approach being prominent examples [8,9]. The three-talk model involves three key steps, including team talk, option talk, and decision talk, which encompass working together with the clinician to describe choices, discuss alternatives, and arrive at informed preferences to make decisions [8]. The Agency for Healthcare Research and Quality proposes the SHARE approach for decision making, which includes a five-step process to seek patient participation, help them choose treatment, assess patient preferences, and reach and evaluate the patient’s decision [9]. SDM becomes more complicated for multimorbid individuals, as managing multimorbidity is not as straightforward as addressing each condition individually, given additional factors such as polypharmacy and increased burden of treatment [10]. 

One systematic review indicated that individuals with multimorbidity are projected to utilize healthcare services at a rate 2.56 times higher than those without multimorbidity [11]. Results from patient-reported outcomes in multimorbid individuals indicated higher levels of anxiety, depression, and worse physical function [12]. The presence of multimorbidity can lead to functional limitations through gradual physical and cognitive decline, which in turn contributes to the development of additional comorbid conditions [13]. Moreover, research shows the highest risk of death, hospitalization, emergency room (ER) visits, and outpatient visits among individuals with both multimorbidity and functional limitations [14]. 

Evaluating the health outcomes, specifically healthcare utilization and mental and physical patient-reported health outcomes, among the multimorbid population is an important area that needs further research. The objective of this study was to determine the association of SDM with healthcare resource utilization and patient-reported health outcomes among United States (US) adults with multimorbidity. 

## 2. Materials and Methods

Data were utilized from the 2020 Medical Expenditure Panel Survey (MEPS) for this cross-sectional study. MEPS is a publicly available, nationally representative dataset of noninstitutionalized US residents. The MEPS household component employs an overlapping panel design to gather data from individual household members through multiple interviews conducted over two consecutive calendar years. The collected data encompass weighted and unweighted frequencies of various variables, including socio-demographic features, health status, medical service utilization, care accessibility, patient-reported healthcare experiences and outcomes, as well as healthcare expenses [15].

The MEPS survey was implemented using three components: the Household Component (HC), the Insurance Component (IC), and the Medical Provider Component (MPC). The HC consists of five interview rounds over two years, using data from about 15,000 households annually, which are subsampled from the previous year’s National Health Interview Survey (NHIS). For the HC, interviews are conducted at the family or household unit level. The IC and MPC surveys collect data from employers and medical providers, respectively [16].

Eligible participants for this study were US adults (18 years or older) with two or more comorbidities (i.e., multimorbidity) who were alive for the entirety of 2020. The list of medical conditions included in the multimorbidity variable was derived from the MEPS list of priority enumeration variables, which includes high blood pressure, heart disease (including coronary heart disease, angina, myocardial infarction, and other unspecified heart disease), stroke, emphysema, chronic bronchitis, high cholesterol, cancer, diabetes, joint pain, arthritis, and asthma. A new variable for multimorbidity was created for this analysis by combining the above-mentioned conditions to determine the eligible population. 

The predictor variable was SDM, categorized as optimal versus not optimal. SDM was determined based on an algorithm of responses to three SDM-related MEPS items that investigated whether the provider helped patients decide on treatments, asked patients about other treatments, and explained all treatment options to patients. A composite metric was derived from these three questions. The response to the first question used a 4-point Likert scale, and the other two questions had binary responses. The responses to the three questions were combined to generate two categories of SDM. For the question that asked if the provider helped patients decide treatments, the responses were assigned scores of “0 = never, 1 = sometimes, 2 = usually, 3 = always”. For the other two questions with binary responses, “1 = yes and 0 = no” was the assigned score for each question. The response of each participant was categorized as optimal or not optimal based on the resultant sum of scores from this metric (5 for optimal SDM and 0–4 for not optimal SDM; Table 1). 

The outcome variables were measures of healthcare resource utilization and self-reported patient outcomes. Healthcare resource utilization variables included the annual number of office-based visits, outpatient visits, ER visits, and inpatient discharges. Healthcare resource utilization variables were categorized as “one or more” versus “none” for response measurement. Patient-reported outcomes were collected through a self-administered Veterans RAND 12 Item Health Survey (VR-12©) in MEPS. VR-12 comprises 12 items used to measure health-related quality of life, estimate disease burden, and evaluate disease-specific implications [17]. The MEPS data include two variables that provide summary scores for the physical and mental components of the 12 patient-reported outcome variables. These scores were converted into dichotomous categories using a median split. 

Differences between the two SDM categories were compared by chi-squared analysis. Unadjusted multivariate logistic regression was performed to assess the association of SDM with healthcare resource utilization and patient-reported outcome variables. Adjusted multivariate logistic regression accounted for demographic characteristics, including age, sex, race, ethnicity, marital status, education status, functional limitation, employment status, and income status, and was conducted for each of the four healthcare resource utilization and the two patient-reported outcome variables (physical and mental components). A supplementary analysis was conducted to assess the association between SDM and each of the 12 constituent items from the patient-reported outcomes (Appendix A). These results were reported as odds ratios (ORs) with a 95% confidence interval (CI). All statistical analyses were performed using SAS (https://www.sas.com/zh_cn/software/stat.html, SAS Institute Inc., Cary, NC, USA). The analysis maintained the complex survey data and was weighted to produce nationally representative estimates. 

## 3. Results

The study sample consisted of 3600 MEPS participants based on the eligibility criteria, with a weighted population of 52,154,473. Among these 3600 participants, 2685 participants reported optimal SDM compared to 915 participants reporting not optimal SDM. 

Table 2 describes the sociodemographic characteristics of study participants based on optimal versus not optimal SDM. All characteristics showed significant differences between individuals who reported optimal versus not optimal SDM, except for sex and ethnicity. The optimal SDM category included a higher number of participants aged 18–64, predominantly white, married, with higher levels of education, no physical or mental limitations, and moderate to high income compared to the not optimal SDM category.

Table 3 describes the univariate and multivariate association of SDM with healthcare resource utilization variables. In the univariate analysis, individuals who reported optimal SDM had increased odds of utilizing at least one office, outpatient, and ER visit annually compared to the not optimal SDM group among multimorbid US adults. In the multivariate regression analysis, after adjusting for age, sex, race, ethnicity, marital status, education status, any limitation, employment status, and poverty status, optimal SDM was statistically associated with more than one annual outpatient visit, ER visit and inpatient discharge. However, at least one office visit was no longer statistically significant in the multivariate analysis.

Table 4 describes the univariate and multivariate association of SDM with the patient-reported outcomes variables. No significant association was found between optimal SDM and either the physical health component or the mental health component of the patient-reported outcomes variables. 

In the supplementary analysis that investigated the association between optimal SDM and each of the 12 patient-reported outcome variables, the univariate analysis showed that individuals who reported optimal SDM had lower odds of reporting limitations for the statement “During a typical day, limitations in climbing several flights of stairs activities”. However, this association was no longer detected in the multivariate analysis. In the multivariate analysis after adjusting for age, sex, race, ethnicity, marital status, education status, any limitation, employment status, and poverty status, the variable “During past 4 weeks, as a result of physical health, limited in kind of work or other activities” was significantly associated with optimal SDM. The other patient-reported outcome variables showed no significant association with optimal SDM in the univariate or multivariate analyses (Appendix A). All multivariate models had a Wald statistic of *p* < 0.05 and a c-statistic value ranging from 0.61 to 0.81, indicating good discriminatory power of the models.

## 4. Discussion

The primary aim of this study was to determine whether SDM is associated with healthcare resource utilization and patient-reported outcomes in U.S. adults with multimorbidity. We assessed SDM in this study based on three items that demonstrated patient participation in decision making, as mentioned in the Section 2 of this paper. 

SDM integrates patient preferences, evidence-based medicine, and the expertise of healthcare professionals, and it is regarded as the gold standard in medical decision-making [18]. The results of this study showed that, among U.S. adults with multimorbidity, optimal SDM was associated with having at least one annual outpatient visit, as well as ER visits and inpatient discharges, in the multivariate analysis. For patient-reported outcomes, optimal SDM was significantly associated with limitations in work/activities due to physical health in the past months. The observed outcomes can be attributed to the specific target population of patients with multimorbidity, who tend to require greater utilization of healthcare resources due to their conditions [19]. 

Compared to the management of a single chronic disease, the management of multiple chronic conditions poses greater complexity [1]. Treatment for individuals with multimorbidity often requires increased utilization of primary care, specialist physician services, higher medication usage, more frequent visits to ERs, and a greater number of hospital admissions [20]. While some studies have reported a decrease in healthcare resource utilization with optimal SDM [21,22], our findings suggest that increased utilization of healthcare resources in the context of SDM may indicate that care is more closely aligned with patient needs and preferences, potentially leading to better long-term outcomes and higher patient satisfaction. Similar findings to those of this study have been reported in the extant literature. A scoping review on healthcare for older adults with multimorbidity found high healthcare resource utilization and higher costs as adverse healthcare outcomes among this patient population [23]. A systematic review and meta-analysis on the costs of multimorbidity concluded that there is an increased economic burden on the health system due to multimorbidity, with outpatient, inpatient, emergency care, and drugs being the most frequently reported cost factors in the studies evaluated [19]. Other studies have confirmed that multimorbidity is linked to higher healthcare resource utilization. For instance, in the systematic review by Soley-Bori et al. on a population with comorbidities, the results showed that multimorbidity was associated with increased total cost and healthcare utilization [11]. In 2017, a study found that the number of chronic conditions people have is directly proportional to their usage of various healthcare services. In addition, these patients have higher healthcare spending [24]. Another literature review observed decreased quality of life and increased healthcare utilization among the seven common challenges for individuals with multiple chronic conditions [25].

Patient-reported outcomes are tools that measure health outcomes from the patient’s perspective, allowing healthcare professionals to understand the patient’s preferences and facilitating a more collaborative approach to healthcare decisions between the patient and the healthcare professional [26]. However, the current study found no statistically significant association between SDM and patient-reported outcomes except for one measure: physical health limitations on the kind of work or other activities performed by the individual. The reason for this significant association may be attributed to the broad nature and construct of the item relative to other items in the category of physical component summary, resulting in significant responses. A study evaluating the association of SDM with patient experience, defined as the patient rating of the provider and whether a patient would recommend the provider, found a modest correlation between the two variables. The study concluded that since increased satisfaction with the healthcare provider led patients to report improved outcomes, there is a need to investigate the two domains of patient-reported outcomes and patient experience with the provider, as they did not find a strong correlation between the two measures [27]. One possible explanation for no association of SDM with patient-reported outcomes in our study could be the lack of a uniform definition for SDM, i.e., different definitions of SDM can lead to different findings, while variations in weighting and scoring systems for SDM could further influence the association with desired outcomes. Further research using different datasets with alternative definitions of SDM may be warranted to investigate this phenomenon further. 

### Limitations

The study is subject to the inherent limitations of a retrospective analysis. For instance, MEPS variables are based on self-reported data so there is a possibility of recall bias. We cannot ascertain a temporal relationship between SDM and healthcare resource utilization or patient-reported outcomes, as assessments for both were performed simultaneously. The findings of our study must be interpreted in the context of the MEPS database, as SDM was measured with three items that may not accurately capture SDM. Patient-reported outcomes were measured via responses to 12 questions in a self-administered survey, which calculated summary scores for physical and mental health. The measurement of healthcare resource utilization was dichotomized. This arbitrary threshold for dichotomizing healthcare resource variables can be a limitation in the interpretation of results. Finally, MEPS does not specify whether SDM responses pertain to a single clinician, primary care, or specialized medical consultation. Despite adjusting for several variables in the analysis, there is a possibility that the presence of known and unknown residual confounding variables influenced our findings. All relevant variables were employed from the dataset, thus this is an inherent limitation of using secondary datasets. Potential confounders like healthcare behaviors or BMI were not included in the model, as our focus was on the impact of SDM on healthcare resource utilization and patient-reported outcomes, which are less directly influenced by these clinically oriented variables. Future analyses could benefit from incorporating a broader range of confounders available in other data sources, as well as employing more advanced statistical techniques, such as propensity score matching, to further mitigate the impact of unmeasured confounding variables. Given the limitations related to the subjective measurement of SDM and patient-reported outcomes, investigating the association between SDM and healthcare metrics using additional databases with different variables defining SDM and patient-reported outcomes may be useful in further evaluating the relationship in populations with multimorbidities. Other studies with similar objectives using different designs and methodologies may produce varying results depending on the data characteristics and the analytical approach taken. Future research should aim to corroborate these findings using alternative definitions, data sources, and analytical frameworks.

## 5. Conclusions

In conclusion, this study showed an association between optimal SDM and increased healthcare resource utilization, perhaps due to informed patients proactively seeking regular check-ups and addressing health concerns promptly. This active engagement, driven by trust and a comprehensive understanding of healthcare options, might result in more frequent office, outpatient, and emergency room visits. The end goal is better-aligned care with individual needs and patient satisfaction. Optimal SDM was not significantly associated with patient-reported outcomes in this analysis except for one item, which inquired about limitations in work or other activities as a result of physical health. Further research is needed to evaluate the impact of SDM on healthcare resource utilization and patient-reported outcomes, especially among populations with multiple chronic conditions. While previous studies have generally shown increased healthcare resource utilization among multimorbid populations, future research should explore the impact of SDM using diverse data sources, alternative definitions of SDM, and different analytical approaches to confirm or challenge our findings.

## Figures and Tables

**Table 1:** Shared decision-making variables in the Medical Expenditure Panel Survey (MEPS) dataset.

Variable	Description	Response Option	Score
DECIDE42	Provider asks person to help make decisions between choice of treatments	Never	0
		Sometimes	1
		Usually	2
		Always	3
TREATM42	Provider usually asks about prescription medications and treatments other doctors may give them	Yes	1
		No	0
EXPLOP42	Provider presents and explains all options	Yes	1
		No	0

**Table 2:** Socio-demographic characteristics of United States adults (≥18 years) with multimorbidity (≥2 chronic conditions) stratified by optimal and not optimal shared decision making.

Variables	Optimal Shared Decision Making Weighted % (95% CI)	Not Optimal Shared Decision Making Weighted % (95% CI)	*p*-Value
Age			
18–64	53.2 (50.3, 56.1)	47.6 (43.5, 51.6)	0.01
≥65	46.8 (43.9, 49.7)	52.4 (48.4, 56.5)	
Sex			
Male	46.3 (44.3, 48.3)	45.8 (41.9, 49.6)	0.75
Female	53.7 (51.7, 55.7)	54.2 (50.3, 58.0)	
Race			
White	84.2 (82.1, 86.3)	78.5 (74.2, 82.7)	<0.01
Other	15.8 (13.7, 17.9)	21.5 (17.3, 25.8)	
Ethnicity			
Hispanic	8.7 (6.8,10.6)	7.4 (5.3, 9.5)	0.29
Non-Hispanic	91.3 (89.4, 93.2)	92.6 (90.5, 94.7)	
Marital status			
Married	60.2 (57.8, 62.7)	51.6 (47.5, 55.6)	<0.01
Other	39.8 (37.3, 42.2)	48.6 (44.4, 52.5)	
Education status			
Up to and including high school	37.2 (34.8, 39.7)	48.5 (44.2, 52.7)	<0.01
More than high school	62.8 (60.3, 65.2)	51.5 (47.3, 55.8)	
Any limitation			
Yes	39.4 (36.9, 41.8)	45.8 (41.9, 49.5)	0.01
No	60.6 (58.2, 63.1)	54.2 (50.5, 57.9)	
Employment status			
Employed	47.6 (45.1, 50.1)	40.3 (36.4, 44.7)	<0.01
Unemployed	52.4 (49.9, 54.9)	59.7 (55.6, 63.9)	
Income/poverty status			
Poor/near poor/low	24.4 (21.9, 26.8)	29.0 (24.9, 33.2)	0.03
Moderate/high	75.6 (73.2, 78.0)	70.6 (66.8, 75.1)	

This analysis was based on an unweighted sample of 3600 United States adults aged ≥18 years with multimorbidity who were alive for the full 2020 calendar year (optimal shared decision-making *n* = 2685; not optimal shared decision-making *n* = 915). Differences between groups were assessed using a chi-squared test. 95% CI = 95% confidence interval.

**Table 3:** Unadjusted and adjusted association of optimal (versus not optimal) shared decision-making with healthcare resource utilization among United States adults with multimorbidity.

	Healthcare Resource Utilization Variables			
	Office Visits (At Least One versus None) None = Ref	Outpatient Visit (At Least One versus None) None = Ref	Emergency Room Visits (At Least One versus None) None = ref	Inpatient Discharges (At Least One versus None) None = Ref
Optimal Shared Decision Making				
Unadjusted OR (95% CI)	1.495 (1.003, 2.228) *	1.218 (1.021, 1.453) *	1.396 (1.065, 1.830) *	1.264 (0.931, 1.715)
Adjusted OR (95% CI)	1.348 (0.929, 1.956)	1.227 (1.027, 1.467) *	1.553 (1.170, 2.063) *	1.437 (1.052, 1.963) *

OR = odds ratio. 95% CI = 95% confidence interval. Ref = reference group. * Indicates significant association.

**Table 4:** Unadjusted and adjusted association of optimal (versus not optimal) shared decision-making with patient-reported outcomes among United States adults with multimorbidity.

	Patient-Reported Outcomes	
	Physical Health Component Summary Score (Above Median Score versus Median Score or Below) Median Score or Below = Ref	Mental Health Component Summary Score (Above Median Score versus Median Score or Below) Median Score or Below = Ref
Optimal Shared Decision Making		
Unadjusted OR (95% CI)	1.139 (0.932, 1.391)	0.882 (0.715, 1.087)
Adjusted OR (95% CI)	0.952 (0.757, 1.198)	0.886 (0.714, 1.098)

OR = odds ratio. 95% CI = 95% confidence interval. Ref = reference group.

## Data Availability

Dataset available on request from the authors.

## Appendix A

**Table A1:** Unadjusted and adjusted association of optimal (versus not optimal) shared decision-making with each patient-reported outcome among United States adults with multimorbidity.

Patient-Reported Outcomes	Optimal Shared Decision Making	
	Unadjusted OR (95% CI)	Adjusted OR (95% CI)
General health today		
Excellent/very good/good	1.052 (0.863, 1.282)	0.856 (0.671, 1.093)
Fair/poor	Ref	Ref
During a typical day, limitations in moderate activities		
Limited a lot/little	0.855 (0.717, 1.020)	1.074 (0.864, 1.335)
Not limited	Ref	Ref
During a typical day, limitations in climbing several flights of stairs activities		
Limited a lot/little	0.801 (0.655, 0.979) *	0.980 (0.778, 1.235)
Not limited	Ref	Ref
During past 4 weeks, as a result of physical health, accomplished less than would like		
All/most/some/little	0.907 (0.761, 1.080)	1.095 (0.877, 1.366)
None	Ref	Ref
During past 4 weeks, as a result of physical health, limited in kind of work or other activities		
All/most/some/little	0.990 (0.823, 1.190)	1.272 (1.011, 1.601) *
None	Ref	Ref
During past 4 weeks, as a result of emotional problems, accomplished less than would like		
All/most/some/little	1.130 (0.935, 1.366)	1.208 (0.975, 1.497)
None	Ref	Ref
During past 4 weeks, as a result of emotional problems, did work or other activities less carefully than usual		
All/most/some/little	1.106 (0.877, 1.394)	1.218 (0.939, 1.581)
None	Ref	Ref
During past 4 weeks, pain interfered with normal work outside the home and housework		
Little/moderate/quite a bit/extreme	1.047 (0.868, 1.263)	1.234 (0.999, 1.524)
Not at all	Ref	Ref
During the past 4 weeks, felt calm and peaceful		
A good bit/most/all of the time	0.952 (0.766, 1.182)	0.954 (0.764, 1.192)
Some/little/none of the time	Ref	Ref
During the past 4 weeks, had a lot of energy		
A good bit/most/all of the time	0.990 (0.816, 1.202)	0.877 (0.706, 1.090)
Some/little/none of the time	Ref	Ref
During the past 4 weeks, felt downhearted and blue		
A good bit/most/all of the time	0.955 (0.697, 1.307)	0.993 (0.711, 1.386)
Some/little/none of the time	Ref	Ref
During the past 4 weeks, physical health or emotional problems interfered with social activities		
All/most/some/little of the time	1.097 (0.897, 1.341)	1.202 (0.958, 1.507)
None of the time	Ref	Ref

OR = odds ratio. 95% CI = 95% confidence interval. * Indicates significant association.
